# Risk factors for revision of total knee arthroplasty: a scoping review

**DOI:** 10.1186/s12891-016-1025-8

**Published:** 2016-04-26

**Authors:** L.L. Jasper, C. A. Jones, J. Mollins, S. L. Pohar, L. A. Beaupre

**Affiliations:** Department of Physical Therapy, University of Alberta, Rm 2-50 Corbett Hall, Edmonton, AB T6G 2G4 Canada; Alberta Health Services, Edmonton, Canada; Canadian Agency for Drugs and Technologies in Health, Ottawa, Canada

**Keywords:** Total knee arthroplasty, Revision, Failure, Risk factor, Scoping review

## Abstract

**Background:**

In spite of the increasing incidence of total knee arthroplasties (TKA), evidence is limited regarding risk factors for revision. The objective of this scoping review was to identify and assess demographic, surgical and health services factors that may increase the risk for revision surgery following TKA.

**Methods:**

A scoping review was undertaken following an electronic search in MEDLINE (1990 to December 2013), CINAHL (to December 2013), EMBASE (1990 to December 2013) and Web of Science (1990 to December 2013).

**Results:**

Of the 4460 articles screened, 42 were included of which 26 articles were based on registry data. Increased risk of revision was associated with demographic factors (younger age, African American), surgical factors related to the primary TKA (uncemented components, implant malalignment, increased surgery duration), and health services (low volume hospitals).

**Conclusions:**

Identifying emerging trends in characteristics of those requiring revision following TKA can help identify those at risk and allocate appropriate resources. Further primary clinical articles on risk factors for revision of TKA are necessary to ensure maximal function and lifespan following TKAs.

**Electronic supplementary material:**

The online version of this article (doi:10.1186/s12891-016-1025-8) contains supplementary material, which is available to authorized users.

## Background

The effectiveness of total knee arthroplasty (TKA) in relieving pain and improving function has been well documented [1, 2]. TKA is considered a cost effective and efficacious treatment for patients with end stage knee osteoarthritis who experience severe pain, activity limitations and for whom conservative treatment is unsuccessful [3–5]. With more than 700 000 primary TKAs performed annually in the USA, estimates of TKA are projected to increase to 673 % by 2030 in the USA. The large demand for TKA is primarily related to the aging population, the obesity epidemic and technical advancement of the surgical procedure [6–8]. The longevity of implants is typically greater than 10 years with 32,700 revisions performed annually in the USA. Significant demand for primary TKA will correspond to a growing demand for revisions of TKA which are projected to increase by 601 % from 2005 to 2030 [6].

Revisions for TKA pose unique challenges as revision surgery is a more complex surgery than a primary TKA with increased complication and mortality rates [9–11]. Identifying emerging trends in characteristics of those requiring revision following TKA can help identify those at risk and allocate appropriate resources. Several articles have identified risk factors for revision surgery of TKA yet, to our knowledge, the synthesis of these findings have not been documented. A more comprehensive understanding of the potential risk factors for revision of TKA will provide important knowledge for surgeons and patients. The objective of this scoping review was to identify and assess demographic, surgical and health services factors that lead to increased risk of revision surgery following TKA.

## Methods

A scoping review of the literature was undertaken to identify and assess relevant evidence given the limited existing evidence on revisions of TKA. Inclusion criteria consisted of studies that comprised a) adult patients who received primary TKA and received a subsequent revision, b) comparative groups or risk-adjusted analyses, and c) at least 20 or more revision cases. Cohort and case control articles were included while descriptive studies and randomized controlled trials comparing specific interventions were excluded. Articles which included hemiarthroplasty, primary TKA used to stabilize a fracture or management of bone pathology or malignancy, simultaneous bilateral TKAs, and patellofemoral arthroplasty were excluded. Revisions for all reasons were included except revisions occurring in the first three months due to sepsis. Ethics was not obtained for this study as the study was a retrospective scoping review that did not involve any individual data or identifying information. In discussion with our Health Research Ethics Board at the University of Alberta, we do not require ethics for review.

### Data sources and search strategies

A search strategy was developed and implemented by a health sciences librarian for 4 databases: Medline (1990-Dec 2013; includes in-process & other non-indexed citations), EMBASE (1990-Dec 2013), CINAHL (1990-Dec 2013), and Web of Science (1990-Nov 2012) (Additional file 1). Date (1990–2013) and language (English) restrictions were applied to the searches. The decision to restrict the search to English articles was based on findings from systematic research evidence that reported no empirical evidence of bias was seen if papers written in languages other than English (LOE) were excluded [12]. The search included an extensive list of subject headings and keyword terms for 3 concepts: 1) hip or knee arthroplasty, 2) revision surgery, and 3) prognosis (see Additional file 1). Total hip arthroplasty articles were included in the search because we did not want to inadvertently exclude articles that reported both total hip and total knee arthroplasties. Case articles or case reports were removed along with conference abstracts. This initial search yielded many non-relevant papers so an additional search string was added to increase the relevancy of the results (by including certain terms in either the title or marked as the most important subject headings). A “relevancy forcing search set” was performed to ensure that all relevant papers were captured. All duplicate citations were removed.

### Study selection

To ensure consistency with screening of title and abstract, 20 citations were independently reviewed by both reviewers (LJ & SP) using a standardized form based on broad criteria including population intervention, comparison, outcome and study design. The remaining citations were then independently screened for relevance.

If a citation was selected by either reviewer, the full-text article was obtained for further review. Full-text articles were further screened for selection using a standard study selection form, based upon the predetermined inclusion criteria. The study selection form was initially piloted on a sample of 20 articles to ensure that the selection criteria were applied consistently across reviewers. Relevant full-text articles were then reviewed by one of the two reviewers using standardized inclusion and exclusion criteria. Disagreement of article inclusion was resolved through consensus between reviewers or through third party adjudication if the reviewers did not arrive at consensus. Full-text papers were included only if consensus was achieved by reviewers. For those articles selected for full review, data were extracted by one reviewer (LJ) and verified by a second reviewer (LB or AJ). The first 20 full text articles reviewed by both reviewers had excellent agreement (Kappa value 0.96, *p* < 0.0001). All selected articles were included in data synthesis regardless of methodological quality. Inconclusive findings and gaps in the literature were identified. A narrative description of the included articles was completed and potential patterns identified in terms of targeted behaviors, study outcomes, and intervention effectiveness.

#### Quality assessment

The Oxford Level of Evidence was used to evaluate the quality of selected full-text articles [13], and has been recommended to determine a hierarchy of the best evidence [14]. SIGN guidelines were also used to assess study quality through completion of their cohort checklist including items such as subject selection, assessment, confounding and statistical analysis [15].

## Results

**Fig. 1:**
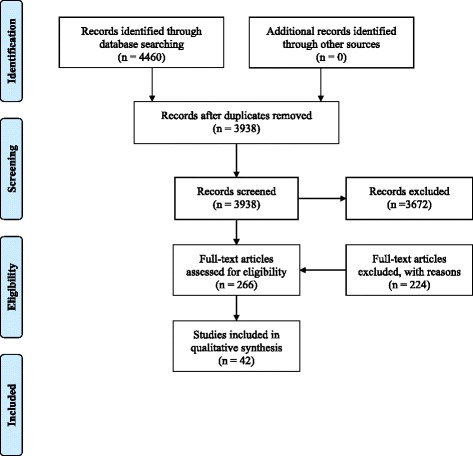
PRISMA flow diagram

Of the 4460 articles identified through the search strategies, 266 articles remained after the abstracts were screened for eligibility. After full text review, 42 articles met the inclusion criteria for the review (see Fig. 1). Twenty-six (62 %) articles were based on registry or insurance databases of which 12 were based on Nordic registries and 11 from American databases (see Additional file 2).

All articles were prognostic retrospective articles with level III quality except for one which was a level II prognostic prospective study [13, 16]. Using the SIGN guidelines, 31 articles were regarded of *acceptable* quality and 11 articles were deemed *poor* quality often due to incomplete reporting of multivariate analyses (see Additional file 2) [15].

Of the 34 (81 %) articles that reported mean follow-up from the primary TKA, six articles reported 10 year survival rates and two articles reported 20 year survival rates (see Table 1). While survival rates of the primary TKA were consistently high at 10 years ranging from 89.5 to 98.6 % [17–19, 20, 21, 42], 20 year survival rates were expectedly lower at 78–99 % [19, 23].

**Table 1:** Survival rates at 10 and 20 Years^a^

Author(s), year	Duration of follow-up (yrs)	Index procedure (n)	Revision (n)	Survival rate (%, CI)
Badawy M. et al., 2013 [42]	10	26,698	1169	Low hospital volume^b^: 92.5 %, 95 % CI 91.5 to 93.4 High hospital volume: 95.5 %, 95 % CI 94.1 to 97.0
Gothesen, Ø. et al., 2013 [17]	10	17,782	NR	89.5 to 95.3 %, CI- NR
Himanen, A. et al., 2007 [20]	10	751	37	Prosthetic moulded component: 94.4 %, 95 % CI 90.4 to 96.7 Prosthetic modular component: 93.6 %, 95 % CI 89.7 to 96.0
Jämsen, E. et al., 2013 [21]	10	53,007	1919	94.5 %, 95 % CI 94.1 to 94.8
Rand, J. et al., 2003 [19]	10	11,606	NR	91 %, 95 % CI 90 to 91 %
Vessely, M. et al., 2006 [18]	10	1000	45	98.6 %, 95 % CI 97.8 to 99.4
Fang, D. et al., 2009 [23]	20	6070	51	99 %, CI - NR
Rand, J. et al., 2003 [19]	20	11,606	NR	78 %, 95 % CI 74 to 81 %

^a^See Additional file 2 for further detail. Not reported in publication, NR

^b^Low hospital volume is 1–24 TKA performed per year; High hospital volume is ≥150 TKA performed per year

### Demographic risk factors

#### Sex

While all articles reported sex, the association of sex and TKA revision was only examined in 10 articles (see Table 2). Inconsistent findings were reported in that males had a higher risk of revision surgery than females in 5 articles (see Table 2), females had a higher risk of revision (HR 1.513, 95 % CI 1.116 to 2.051) in one article, based on American registry data, [24] and four articles, from different countries, did not find a significant association between sex and TKA revision [16, 18, 25–26].

**Table 2:** Sex and adjusted risk of revision^a^

Author(s)/year	Control	Hazard ratio (CI)
Blum, M. et al., 2013 [29]	Female	0.81, 95 % CI 0.71 - 0.92, *p* < 0.01
Fehring, T. et al., 2004 [52]	Male	2.771, 99 % CI 1.662 - 4.620, *p* < 0.0001
Harrysson, O. et al., 2004 [43]	Male	1.64, 95 % CI 1.23 - 2.18, *p* = 0.0007
Rand, J. et al., 2003 [19]	Male	1.6, 95 % CI 1.4 - 2.0, *p* < 0.0001
Schrama, J. et al., 2010 [30]	Female	0.67, 95 % C I 0.47 - 0.88
Stiehl, J. et al., 2006 [24]	Female	1.513, 95 % CI 1.116 - 2.051

^a^See Additional file 2 for further detail

#### Age

Among the 15 articles that examined age as a risk factor, 13 articles reported that revision rates decreased with older age (see Table 3).

**Table 3:** Age and adjusted risk of revision^a^

Author(s)/Year	Age	Hazard ratio (95 % CI) unless otherwise stated
Bini, S. et al., 2013 [31]	>55 years	0.43, 95 % CI 0.27 to 0.67, *p* < 0.001
Blum, M. et al., 2013 [29]	18–64 years vs. 65+ yrs	2.30, 95 % CI 1.96 to 2.69, *p* < 0.0001
Bordini, B. et al., 2009 [32]	Age at surgery (per year)	1.05, 95 % CI 1.03 to 1.06, *p* = 0.0001
Fehring, T. et al., 2004 [52]	Age at surgery (per year)	0.953, 99 % CI 0.932 to 0.975, *p* < 0.0001
Gioe, T. et al., 2004 [53]	Age <70 year	0.46, 95 % CI.0.33 to 0.64, *p* < 0.001
Harrysson, O. et al., 2004 [43]	Older patients (≥60 year) Revision Attributable to Any Reason Revision Attributable to Loosening of Components	0.49, 95 % CI 0.38 to 0.62, *p* < 0.0001 0.41, 95 % CI 0.27 to 0.62, *p* < 0.0001
Julin, J. et al., 2010 [35]	Age ≤ 55 years: Revision for reasons other than infection Revision for any reason	2.9, 95 % CI 2.3 to 3.6 2.4, 95 % CI 2.0 to 3.0 Age 56–65 years
	Age 56–65 years: Revision for reasons other than infection Revision for any reason :	1.7 95 % CI 1.4 to 2.0 1.5, 95 % CI 1.3 to 1.7
Kreder, H. et al., 2003 [25]	Younger age per 10 year: At 1 year after revision At 3 years after revision	OR 0.77, 95 % CI 0.67 to 0.89 OR 0.70, 95 % CI 0.66 to 0.81
Lygre, S. et al., 2011 [37]	Age >70 year vs. <60 year	0.4, 95 % CI 0.3–0.4, 0 < 0.001
Namba, R. et al., 2013 [28]	Age (increasing 10 year increments)	0.62, 95 % CI 0.57 to 0.67, *p* < 0.001
Namba, R. et al., 2012 [27]	Age (increasing 10 year increments)	0.64, 95 % CI 0.58 to 0.70, *p* < 0.001
Rand, J. et al., 2003 [19]	Age 56–70 year vs. ≤55 years Age >70 year vs. ≤55 years	0.7, 95 % CI 0.5 to 0.9, *p* < 0.01 0.5, 95 % CI 0.3 to 0.6, *p* < 0.0001
Stiehl, J. et al., 2006 [24]	Younger patients (for every yr increase)	0.979, 95 % CI 0.968 to 0.989

^a^See Additional file 2 for further detail

#### Race

Race was examined in 3 American articles, of which 2 were based on the same registry [27, 28]. African American patients had a higher risk for revision than Caucasian patients (HR 1.73, 95 % CI 1.33 to 2.25, *p* < 0.001; HR 1.82, 95 % CI 1.33 to 2.48, *p* < 0.001; HR 1.39, 95 % CI 1.08 to 1.80, *p* = 0.01) and represented 5.5 and 8.4 % of the patient population reported in these registries. [27–29].

### Medical risk factors

#### Primary diagnosis

Although the majority of patients undergoing TKA were diagnosed with osteoarthritis, 4 articles specifically examined diagnosis and its potential association with TKA revision with mixed results [19, 24, 25, 30]. Two large registry articles reported differing results with inflammatory arthritis having a greater and lesser risk for revisions than patients with osteoarthritis (HR 1.6, 95 % CI 1.06 to 2.38 and HR 0.5, 95 % CI 0.3 to 0.7, *p* < 0.001) [19, 30]. A clinical study of 4743 patients found that OA or post-traumatic arthritis had a greater risk of revision than RA (HR 1.51, 95 % CI 1.116 to 2.051) [24]. Further, in a clinical sample of 14352 patients, Kreder et al. reported no significant association between the diagnosis of OA and risk for revision [25].

#### Comorbidities

Eleven articles specifically looked at the effect of comorbidities examining both total number of conditions and specific conditions (see Additional file 2). Jamsen et al. found that risks increased if there were one or more of the comorbidities identified (HR 1.23, 95 % CI 1.16 to 1.30) [21]. Alternately, Kreder et al. did not find a significant association between the presence of comorbidities and revision following TKA [25].

When looking at comorbidities associated with OA, obesity, cardiac disease and diabetes were at high risk of revision. Two American TKA registries reported increased risk of revision for patients with a higher BMI (BMI 30–35 kg vs <30 kg HR 1.48, 95 % CI 1.00 to 2.19 and BMI ≥35 kg/m2 vs. <30 kg/m2 h 0.78, 95 % CI 0.63 to 0.96, *p* = 0.020) [28, 31]. However, 3 other articles did not find a significant relationship between BMI and risk for TKA revision [18, 20, 32].

The presence of cardiac conditions at time of the primary TKA increased the risk of revision including hypertension with early revision (0 – 5 years) (HR 1.14, 95 % CI 1.01 to 1.29), coronary disease (HR 1.27 95 % CI 1.07 to 1.50) and cardiovascular disease (HR 1.29, 95 % CI 1.14 to 1.45) [21, 33].

Three articles reported an increased risk of revision for the patients with diabetes. Jamsen et al. and Namba both found an association with diabetes and revision (HR 1.27, 95 % CI 1.08 to 1.50 and HR 1.21, 95 % CI 1.04 to 1.41, *p* = 0.014) although Jamsen et al. was examining early revisions [28, 21]. Similarly, King et al. also found the 46 to 55 years and 66 years + diabetic cohorts had increased risk of revision as compared to the non-diabetic cohort (HR 2.9 95 % CI 1.5 to 5.8, *p* = 0.004 and HR 1.5, *p* = 0.0037 respectively) although there was not a significant difference in the 56 to 65 years cohort [34].

### Joint implant factors

#### Fixation

Two articles consisting of 9337 patients from the US found cemented primary TKAs had a protective effect on receiving revision as compared to cementless/hybrid TKAs [16, 19]. Hybrid fixation, in which the proximal component was cementless and the distal component was cemented, also demonstrated a higher risk for revision than cemented TKAs in both US and Norwegian studies [16, 35].

#### Cruciate retaining implants

Cruciate ligament status was reported in several articles with inconsistent findings (see Table 4) [16]. Two large American registry studies reported that posterior stabilized implants had increased risk of revision when compared to posterior cruciate-retaining implants (HR 2.6, 95 % CI 2.1 to 3.5, *p* < 0.0001 and HR = 2.0, 95 % CI 1.67 to 2.5, *p* < 0.001) [19, 36]. Conversely, an American registry study of 1047 patients found ligament status was not significant [16]. Further, Stiehl et al. found both posterior cruciate retaining arthroplasties and bicruciate retaining arthroplasties had increased risk of revision compared to rotating platform (HR 1.552, 95 % CI 1.157 to 2.081 and HR 2.188, 95 % CI 1.454 to 3.294) [24].

**Table 4:** Implant type/technique and adjusted risk of revision^a^

Author(s)/year	Implant type/technique	Reference	Hazard ratio (95 % CI) unless otherwise stated
Abdel, M. et al., 2011 [36]	Cruciate Status:		
	Posterior cruciate-retaining	Posterior cruciate-stabilizing	0.5, 95 % CI 0.4 - 0.6, *p* < 0.001
Rand, J. et al., 2003 [19]	Cruciate Status:		
	Posterior Stabilized	Posterior cruciate-retaining	2.6, 95 % CI 2.1 - 3.5, *p* < 0.0001
	Constrained condylar	Posterior cruciate-retaining	2.1, 95 % CI 0.9 - 4.9, *p* = 0.08
Stiehl J. et al., 2006 [24]	Cruciate Status		
	PCRs	Rotating platform	1.552, 95 % CI 1.157 - 2.081
	BCRs		2.188, 95 % CI 1.454 - 3.294
Gøthesen, O. et al., 2013 [17]	Implant Type:		
	Duracon	Profix	2.6, 95 % CI 1.9 - 3.4, *p* < 0.001
	LCS Classic HR		1.3, 95 % CI 1.0 - 1.6, *p* = 0.017
	LCS Complete		1.5, 95 % CI 1.1 - 1.9, *p* = 0.002
	AGC Universal		1.6, 95 % CI 1.3 - 2.0, *p* < 0.001
Lygre, S. et al., 2010 [37]	Implant Type:		Relative Risk =
	NR Tricon	NR AGC Universal	1.67, 95 % CI 1.24–2.24, *p* = 0.001,
	NR Genesis 1		1.43, 95 % CI 1.14–1.79, *p* = 0.002,
	NR Duracon		1.45, 95 % CI 1.05–1.99, *p* = 002.
	NR Profix		0.66, 95 % CI 0.52–0.82, *p* < 0.001,
	NR e.motion		0.09, 95 % CI 0.02–0.37, *p* = 0.001,
	NR AGC anatomic		0.66, 95 % CI 0.45–0.99, *p* = 0.04,
	PR AGC universal		0.48, 95 % CI 0.27–0.83, *p* = 0.009,
	PR NexGen		0.40, 95 % CI 0.22–0.74, *p* = 0.004.
Namba R. et al., 2013 [28]	Implant Type:		
	Rotate LCS	Fixed PS	2.07, 95 % CI 1.53 - 2.80, *p* < 0.001
	High flexion	Yes versus No	1.76, 95 % CI 1.29 - 2.41, *p* < 0.001
Namba R. et al., 2012 [27]	Implant Type:		
	LCS	Fixed	2.01, 95 % CI 1.41 - 2.86, *p* < 0.001
Inacio M. et al., 2013 [54]	Bearing or inserts:		
	CoCr-HXLPE	CoCr-CPE	NS 1.2, 95 % CI 0.9 - 1.5, *p* > 0.05
	OZ-CPE	C0Cr-CPE	NS 1.4, 95 % CI 0.3 - 5.9, *p* > 0.05

*Abbreviations: RR* relative risk, *NS* not significant, *BCR* bicruciate preservation, *PCR* posterior cruciate retention, *(PR)* patella resurfaced, *(NR)* patella non resurfaced, *LCS* low contact stress, *OZ* oxidized zirconium, *CoCR* cobalt chromium, *CPE* conventional polyethylene, *HXLPE* highly crosslinked polyethylene;

^a^See Additional file 2 for further detail

#### Patellar resurfacing

The articles that specifically examined patellar resurfacing had inconsistent findings. Three articles found that the risk of revision increased when the patella was resurfaced (patella not resurfaced HR 1.4, 95 % CI 1.2 to 1.7, patella resurfaced HR 0.84, 95 % CI: 0.071–1.0, *p* = 0.052, and patellar resurfaced HR 1.814, 95 % CI 1.320 to 2.558 respectively) [24, 35, 37]. Alternately, two articles found that the patellae not resurfaced patellae had higher risks of revision than resurfaced patellae (HR 2.09, 95 % CI 1.07 to 4.06, *p* = 0.03, HR 1.4, 95 % CI 1.2 to 1.7) [27, 35]. Two studies did not find an association between patellar resurfacing and revision significant [20, 38]. One study reported that metal-backed patella were more likely to be revised than all polyethylene patellar components (HR 2.4, 95 % CI 1.9 to 3.1, *p* < 0.0001) [19].

#### Alignment

Malalignment was reported to be a large risk factor for revisions (HR >2.7) in three studies with both varus and valgus malalignment having a greater risk of revision [23, 39, 40]. Two American studies reported an increased risk of revision with varus tibial malalignment (<90°) (HR 10.6, 95 % CI 5.4 to 20.6, *p* < 0.0001; OR 3.0, *p* = 0.04 respectively) [23, 39]. Valgus femoral malalignment also showed an increased risk with ≥8° of valgus (HR 5.1, 95 % CI 2.8 to 9.5, *p* < 0.0001) [40].

#### Bone quality

As bone stock is a key determinant of the type of implant used and possible peri-prosthetic fracture, bone quality is an important surgical consideration. Only one study examined bisphosphonate use and reported a protective effect for risk for revision (HR 0.40, 95 % CI 0.15 to 1.07, *p* = 0.068) [41] recommending its use for those patients with the diagnosis of osteoarthritis.

### Health services

Of the 3 articles that reported hospital volume in Canada, USA and Norway, low volume hospitals had an increased risk for revision of primary TKAs [25, 42, 22]. The definition of low volume, however, varied from less than 25 to less than 50 procedures annually. Further, Harrysson et al. found that the risk of revision decreased when comparing the year of surgery to the previous year (HR 0.92, 95 % CI 0.89 to 0.96, *p* < 0.0001) over a 10 year time period [43].

Length of surgery for the primary TKA was also found to have a significant association with revision risk in TKA primary surgery >240 min (OR 1.34, 95 % CI 1.07 to 1.67, *p* = 0.012) as compared to <240 min, 150 to 180 min (OR 1.31, 95 % CI 1.09 to 1.57, *p* = 0.004) as compared to 120 to 150 min and <90 min (OR 1.47, 95 % CI 1.10–1.95, *p* = 0.008) as compared to 120 to 150 min [44].

## Discussion

We identified 42 articles that reported risk factors for TKA revision using risk-adjusted analyses. Demographic, medical and implant factors were identified as risk factors for revision of TKA ranging from short-term (<5 years) to long-term follow-up (20+ years). Risk factors were derived largely from registry data, which inherently restricts the type of risk factors examined.

Primary TKA has been consistently identified as a successful surgery with high survival rates even at 10 and 20 years post-surgery. Others have reported rates of 1.26 revisions per 100 observed component years for TKA as compared to 1.29 revisions per 100 observed component years for total hip replacements and 3.29 revisions per 100 component years for total ankle replacements [45]. Given the success of the surgery, it has been suggested the focus of research should perhaps shift to patient selection for these procedures to optimize outcomes and health resources [46].

The trend of increasing revision rates will likely increase [46, 47]. This information was especially relevant given that the 45–64 year old cohort is one of the fastest growing demographics [48, 49]. Further, this age cohort demonstrated an increased use of TKA and will require a longer life expectancy for the TKA, an important consideration when planning for future allocation of resources [46, 49]. The increased risk for revision in the younger population must be further examined to determine if it is indeed age that is the risk factor or if age is a proxy for higher activity levels or increased expectations in this younger patient population.

Comorbidities such as diabetes, cardiovascular disease, hypertension, obesity, cancer and lung disease were found to increase the risk for revision. These findings are particularly meaningful given the increasing prevalence of multi-morbidity and the challenge of surgical management of patients with other chronic diseases [50, 51]. Further investigation of management programs of secondary chronic diseases such as hypertension, obesity and diabetes in patients with primary TKA is warranted.

Often heterogeneity was found among the reported results for other risk factors for TKA revision. For example, mixed results were reported regarding sex, primary diagnosis, BMI, patellar resurfacing and implant components suggesting a need for further investigation. Some consensus existed, however, regarding cemented prostheses which had a lower risk of revision than uncemented or hybrid in spite of an initial goal of uncemented fixation to decrease complications associated with aseptic loosening [16, 19, 35]. Increased surgery length and low hospital volumes were also found to negatively affect revision rates which is important information to consider in health resource allocation and planning.

In spite of a wide body of literature published on various surgical factors, many articles were of low quality and few included risk-adjusted analysis. The majority of included articles (41/42) were retrospective prognostic articles limiting the quality of the articles to an Oxford level III. Because the majority of data (26/42) was taken from registry data, the data were often limited to basic demographic information such as age, gender and BMI and did not evaluate pain and functional measures (see Additional file 2). An inherent limitation of these large, population-based registries is that demographic, surgical and health services data over decades have typically been evaluated and do not provide patient-reported outcomes or patient-reported experience measures which are central to clinical outcomes of TKA. Finally, findings were derived from two geographical populations, 26 in the USA and 11 in Nordic countries. External validity to other populations is uncertain because of different healthcare systems and potentially different prostheses.

In spite of an extensive search strategy and a strong systematic approach to undertaking this systematic review, identifying risk factors for revision was challenging because of low revision rates in the first 10 years following surgery. Most articles had follow-up periods of <10 years which reflected high survival rates of TKA. Due to these high survival rates, it can be a lengthy and costly process to undertake studies for the appropriate duration to acquire accurate information on revisions. Another consideration is that many early revisions occurring within 10 years are often related to surgical techniques and few articles made the distinction between early and later revisions. Finally, as TKAs are most often performed on an older population, the development of other chronic conditions and mortality poses a challenge to long-term follow-up.

## Conclusions

Current literature suggests an increased risk for revision following TKA is associated with younger age, greater number of comorbidities, African American race, uncemented components, increased surgery duration, and lower volume hospitals. This scoping review allowed us to identify areas where consistent results were found but also highlight areas with heterogeneous results or insufficient data where further research is required. The findings also demonstrate the need for large scale and high quality investigations examining factors that increase the risk for revision following TKA including patient-reported outcomes and patient-reported experience measures. Given the increasing numbers of TKA procedures and revisions, information on risk factors for revisions following TKAs is necessary for appropriate interventions to be delivered in a timely manner and for the development of effective health care policy.

### Availability of data and materials

The dataset supporting the conclusions of this article is included within the article and its additional files.

## Additional files

Additional file 1: Search Strategy. (DOCX 14 kb) Additional file 2: Characteristics of Included Studies. [16–20, 27–34, 35–37, 38–44, 55–65]. (DOCX 70 kb)
