# Supplementary material for: Risk factors for revision of total knee arthroplasty: a scoping review
Source: BMC Musculoskelet Disord. 2016 Apr 26;17:182. doi: 10.1186/s12891-016-1025-8 (PMC4845333; doi:10.1186/s12891-016-1025-8)
Supplement: Additional file 1: — Search Strategy. (DOCX 14 kb) [file 12891_2016_1025_MOESM1_ESM.docx]

**Supplemental File 1.** Search Strategies in 4 Electronic Databases (MEDLINE, EMBASE, Web of Science, CINAHL) Using Language (English).

**Ovid MEDLINE(R)** In-Process & Other Non-Indexed Citations, Ovid MEDLINE(R) Daily and Ovid MEDLINE(R) 1946 to Present

Date searched: Dec 6, 2013

1. hip joint/ or hip/

2. Knee Joint/ or Knee/

3. "prostheses and implants"/ or joint prosthesis/

4. arthroplasty/ or arthroplasty, replacement/

5. (1 or 2) and (3 or 4)

6. hip prosthesis/ or knee prosthesis/

7. arthroplasty, replacement, hip/ or arthroplasty, replacement, knee/

8. ((total or complete) adj6 (hip or hips or knee or knees) adj6 (arthroplast* or prosthe* or replace* or implant*)).mp.

9. (((total or complete) adj6 joint adj6 (arthroplast* or prosthe* or replace* or implant*)) and (hip or hips or knee or knees)).mp.

10. (TKA or THA or TJA or TKR or TJR or THR).ti.

11. or/5-10

12. Reoperation/

13. Prosthesis Failure/

14. (fail* or revis* or re-operat* or reoperat* or repeat* or reimplant* or reconstruct*).mp.

15. or/12-14

16. 15 and 11

17. ((predict* or rate or risk) adj6 (fail* or revis* or re-operat* or reoperat* or repeat* or reimplant* or reconstruct*)).mp.

18. (survival adj2 rate).mp.

19. (failure* adj2 analysis).mp.

20. (survival or non-survival or failure or prognos* or predict* or risk factor*).ti.

21. prognosis/

22. survival/ or survival rate/ or survival analysis/

23. or/17-22

24. 16 and 23

25. limit 24 to yr="1990 -Current"

26. Epidemiologic studies/

27. exp case control studies/

28. exp cohort studies/

29. Case control.tw.

30. (cohort adj (study or studies)).tw.

31. Cohort analy$.tw.

32. (Follow up adj (study or studies)).tw.

33. (long-term or longterm).ti.

34. (observational adj (study or studies)).tw.

35. (Longitudinal or prospective or Retrospective or Cross sectional).mp.

36. Cross-sectional studies/

37. (regist* or matched-pair* or matched pair*).mp.

38. or/26-37

39. case reports/

40. ((case not (case control or case-control or case series or case-series or case-cohort or case cohort)) adj4 (study or report*)).tw.

41. ((year* old or month* old or day* old or yr* old or y old) adj3 (female or male or child or woman or man or girl or boy or baby)).ab.

42. case report*.jw.

43. or/39-42

44. 38 not 43

45. 25 and 44

46. *arthroplasty, replacement, hip/ or *arthroplasty, replacement, knee/

47. (TKA or THA or TJA or TKR or TJR or THR or cruciate retaining or cruciate substituting or ((hip or hips or knee or knees or joint or regist*) and (total or arthroplast* or prosthe* or replace* or implant*))).ti.

48. 45 and (47 or 46)

49. (resurfacing or hemiarthroplast* or hemi arthroplast*).ti.

50. 48 not 49

**Embase** 1974 to 2013 December 04

Searched: Dec 6, 2013

1. total hip prosthesis/ or total knee replacement/

2. knee prosthesis/ or hip prosthesis/

3. knee arthroplasty/ or hip arthroplasty/

4. (arthroplasty/ or joint prosthesis/) and (knee/ or hip/)

5. ((total or complete) adj6 (hip or hips or knee or knees) adj6 (arthroplast* or prosthe* or replace* or implant*)).mp.

6. (((total or complete) adj6 joint adj6 (arthroplast* or prosthe* or replace* or implant*)) and (hip or hips or knee or knees)).mp.

7. (TKA or THA or TJA or TKR or TJR or THR).ti.

8. or/1-7

9. reoperation/

10. exp prosthesis failure/

11. (fail* or revis* or re-operat* or reoperat* or repeat* or reimplant* or reconstruct*).mp.

12. or/9-11

13. ((predict* or rate or risk) adj6 (fail* or revis* or re-operat* or reoperat* or repeat* or reimplant* or reconstruct*)).mp.

14. (survival adj2 rate).mp.

15. (failure* adj2 analysis).mp.

16. (survival or non-survival or failure or prognos* or predict* or risk factor*).ti.

17. prognosis/

18. long term survival/ or event free survival/ or survival prediction/ or survival factor/ or survival/ or failure free survival/ or survival rate/

19. or/13-18

20. 8 and 12 and 19

21. limit 20 to yr="1990 -Current"

22. clinical study/

23. exp case control study/

24. family study/

25. longitudinal study/

26. retrospective study/

27. prospective study/

28. "randomized controlled trial (topic)"/

29. 27 not 28

30. cohort analysis/

31. (Cohort adj (study or studies)).mp.

32. (Case control adj (study or studies)).tw.

33. (follow up adj (study or studies)).tw.

34. (observational adj (study or studies)).tw.

35. (epidemiologic$ adj (study or studies)).tw.

36. (cross sectional adj (study or studies)).tw.

37. (long-term or longterm).ti.

38. regist*.mp.

39. matched pair*.tw.

40. or/22-27,30-39

41. case report/

42. ((case not (case control or case-control or case series or case-series or case-cohort or case cohort)) adj4 (study or report*)).tw.

43. ((year* old or month* old or day* old or yr* old or y old) adj3 (female or male or child or woman or man or girl or boy or baby)).ab.

44. case report*.jw.

45. or/41-44

46. 21 not 45

47. 40 and 46

48. (resurfacing or hemiarthroplast* or hemi arthroplast*).ti.

49. 47 not 48

50. limit 49 to conference abstract

51. 49 not 50

**Web of Science** (ISI Interface)

Searched: Dec 6, 2013

Databases: SCI-EXPANDED, SSCI, A&HCI

Date limit 1990-2013

#1 TI=(TKA OR THA OR TJA OR TKR OR TJR OR THR)

#2 TS=((total or complete) NEAR/4 joint NEAR/4 (arthroplast* or prosthe* or replace* or implant*)) AND TS=(hip or hips or knee or knees)

#3 TS=((total or complete) NEAR/4 (hip or hips or knee or knees) NEAR/4 (arthroplast* or prosthe* or replace* or implant*))

#4 TS=(TKA or THA or TJA or TKR or TJR OR THR) AND TS=((joint or hip or hips or knee or knees) NEAR/4 (arthroplast* or prosthe* or replace* or implant*))

#5 #1 OR #2 OR #3 OR #4

#6 TS=(fail* or revis* or re-operat* or reoperat* or repeat* or reimplant* or reconstruct*)

#7 TS=((predict* OR rate OR risk OR factor*) NEAR/6 (fail* OR revis* OR reoperat* OR reoperat* OR repeat* OR reimplant* OR reconstruct* OR survival)) OR TS=("survival rate" or "failure analysis" or non-survival or "longterm survival" or "long-term survival") OR TI=(survival or non-survival or failure or prognos* or predict* or factor*)

#8 #5 AND #6 AND #7

#9 TI=(resurfacing or hemiarthroplast* or hemi arthroplast*)

#10 #8 NOT #9

#11 TS=(cohort or follow-up or "long term" or longterm or longitudinal or prospective or retrospective or register or registry or "matched pair*" or "cross sectional" or cross-sectional or observational or case-control or "case control") NOT TS=(case study or (case NEAR/3 report))

#12 #10 AND #11

**CINAHL Plus with Full Text**  (EBSCO Interface)

Searched: Dec 6, 2013

*Search modes: Boolean/Phrase*

S1 ( (MH "Arthroplasty, Replacement, Hip") OR (MH "Arthroplasty, Replacement, Knee") ) OR ( (total or complete) n6 (hip or hips or knee or knees) n6 (arthroplast* or prosthe* or replace* or implant*) ) OR ( ((total or complete) n6 joint n6 (arthroplast* or prosthe* or replace* or implant*)) and (hip or hips or knee or knees) ) OR ( TKA or THA or TJA or TKR or TJR or THR )

S2 (MH "Reoperation") OR ( ( MH "Prosthesis Failure") OR (MH "Equipment Failure") ) OR ( fail* or revis* or re-operat* or reoperat* or repeat* or reimplant* or reconstruct* )

S3 ( (MH "Prognosis") OR (MH "Survival Analysis") OR (MH "Survival") ) OR ( ((predict* or rate or risk) n6 (fail* or revis* or re-operat* or reoperat* or repeat* or reimplant* or reconstruct*)) ) OR ( longterm survival or long-term survival or survival rate or failure analysis or ) OR TI ( urvival or non-survival or failure or prognos* or predict* or factor* )

S4 S1 AND S2 AND S3

S5 TI (resurfacing or hemiarthroplast* or hemi arthroplast*)

S6 S4 NOT S5

S7 ( (MH "Prospective Studies+") OR (MH "Case Control Studies+") OR (MH "Correlational Studies") OR (MH "Cross Sectional Studies") ) OR ( cohort* or observational stud* or longterm or retropective* or long-term or longitudinal or follow-up or cross-sectional ) OR TI regist*

S8 S6 AND S7
