# Supplementary material for: Risk factors for revision of total knee arthroplasty: a scoping review
Source: BMC Musculoskelet Disord. 2016 Apr 26;17:182. doi: 10.1186/s12891-016-1025-8 (PMC4845333; doi:10.1186/s12891-016-1025-8)
Supplement: Additional file 2: — Characteristics of Included Studies. [16–20, 27–34, 35–37, 38–44, 55–65]. (DOCX 70 kb) [file 12891_2016_1025_MOESM2_ESM.docx]

| **Author, Year of Publication, Country** | **Study Design, Oxford Level of Evidence, Joint Registry if applicable,**  **Dates of data collection** | **Study Population** | **Surgical Characteristics** | **Determinant(s)** | **Follow-up Period** | **Reason for Revision** | **Study Quality**  (High Quality ++, Acceptable + or Low Quality 0) | **Results of Study** |
| --- | --- | --- | --- | --- | --- | --- | --- | --- |
| 1. Abdel M et al.  2011  USA^37^ | Prognostic  Level III  Retrospective  Mayo Clinic  Total Joint Database  1988 - 1998 | 8117 TKA  5389 Posterior Cruciate-Retaining (PCR)  2728 Posterior Cruciate-Stabilizing (PCS)  507 revisions  PCR 320  PCS 187  Diagnoses:  OA  Inflammatory arthritis including OA  Posttraumatic Arthritis  Other | 96% Cemented and 4% uncemented implants.  All polyethylene. non-modular, and modular metal-backed tibial components.  Prostheses:  DePuy PFC Cruciate-Retaining (34%)  DePuy PFC Cruciate-Stabilizing PFC (28%)  Smith and Nephew/Richards GenesisI (18%) | Posterior Cruciate-Retaining versus Posterior Cruciate-Stabilizing | Mean 10.2 years (1 day to 20.4 yrs) | Aseptic Revision  Surgery | + | Posterior cruciate-retaining TKAs HR=0.5, 95% CI 0.4 to 0.6, p<0.001 less risk of revision than those with posterior cruciate-stabilizing TKAs  Greater survival was associated with:  - age of more than 70 years (HR 0.35, 95% CI 0.29 to 0.43, p<0.001),  - female sex (HR 0.77, 95% CI 0.67 to 0.91, p<0.007),  - preoperative inflammatory diagnosis (HR 0.7, 95% CI 0.4 to 0.98, p<0/039). |
| 2. Adams A et al.  2013  USA^53^ | Prognostic  Level III  Retrospective  Kaiser Permanente Total Joint Replacement Registry  2001 - 2009 | 40491 patients with TKAs  464 patients with revisions =  345 (1.1%) no diabetes 33(1.7%) controlled diabetes,  31 (1.2%) uncontrolled diabetes  Males:  11840 (36.0%) Nondiabetic  2151 (42.7%) Controlled diabetes  1086 (43.0%) Uncontrolled diabetes  Median age (IQR), yrs:  68 (61-75) Non- diabetes  69 (63-75) Controlled diabetes  67 (61-73) Uncontrolled diabetes  Diagnosis - OA:  31663 (96.2%) Non-diabetes  4907 (97.3%) Controlled diabetes  2462 (97.5%) Uncontrolled diabetes |  | Non-diabetes, Controlled diabetes or uncontrolled diabetes  Age  Sex  BMI  Charlson Comorbidity Index  ASA score  Use of perioperative antibiotic prophylaxis  Use of antibiotic cement | 1 yr | All cause | + | Diabetes and revision OR 1.32, 95% CI 0.99 to 1.76 NS  Uncontrolled diabetes and revision OR 1.03, 95% CI 0.68 to 1.54 NS |
| 3. Badawy M et al.  2013  Norway^22^ | Prognostic  Level III  Retrospective  Norwegian  Arthroplasty Register  1994 – 2010 | 26698 TKAs  1169 revisions  Gender*:  1359 (29%) M < 25 procedures  2324 (31%) M 25 to 49 procedures  3482 (33%) M 50 to 99 procedures  788 (37%) M 100 to 149 procedures  587 (32%) M ≥150 procedures  Mean Age, (range) yrs:  72 yrs (20 – 93) < 25 procedures  72 yrs (22 to 92) 25 to 49 procedures  72 yrs (26 to 96 yrs) 50 to 99 procedures  71 (22 to 92) 100 to 149 procedures  70 yrs (31 to 91) ≥150 procedures | Cemented  Without patellar component  Implants – type and #:  Profix 7002  LCS Complete 5501  AGC 3759  LCS 3511  Genesis 2049  Duracon 1945  Hospital volume:  4685 < 25 procedures  7497 25 to 49 procedures  10551 50 to 99 procedures  2131 100 to 149 procedures  1834 ≥150 procedures | Hospital volume:  Low (1-24)  Medium (25-49)  Medium (50-99)  High (100-149)  High (≥150)  Age  Sex  Diagnosis | NR | All Cause | + | 100-149 procedures per year HR 0.73, 95% CI 0.56 to 0.96, p=0.03 as compared to 1-24 procedures per year  ≥150 procedures per year HR 0.73, 95% CI 0.54 to 1.00, p=0.05 as compared to 1-24 procedures per year    Survival rate 10 yrs :  - 92.5%, 95% CI 91.5 to 93.4 for 1-24 procedures per year  - 95.5%, 95% CI 94.1 to 97.0 for ≥150 procedures per year |
| 4. Berend M et al.  2010  USA^54^ | Prognostic Level III  Retrospective  USA Hospital  1983 - 2006 | 5997 TKAs  3960 patients  53 revisions  1544 (39%) male  2417 (61%) female  Mean age 70 yrs  3802 (96%) OA* | Composite implant thickness ranged 8 to 20 mm | Age  Sex  BMI  Alignment angle  Tibial component position  Polyethylene thickness | Mean 6.8 (2-22.5) yrs | All cause except infection | 0 | Thicker implants (>16 mm) HR 3.2, p=0.0001  Survival rate at 12 yrs thin bearing 98%  Survival rate at 12 yrs thick bearing 94% |
| 5. Bini S et al.  2013  USA^31^ | Prognostic  Level III  Retrospective  Kaiser Permanente  Total Joint Replacement Registry  April 2001 – March 2009 | 16548 TKAs  14274 patients  13835 TKAs pre-coated tray (+PC)  2713 TKAs non-pre-coated tray (-PC)  +PC 138 revisions  -PC 8 revisions  5857 (35%) males  4903 (35.4%) +PC  954 (35.2%) –PC  10690 (65%) females  8931 (64.6%) +PC  1759 (64.8%) –PC  Age (n, %)  <55 yrs 1048 (7.6) +PC  <55 yrs 196 (7.2) -PC  $\geq$55 yrs 12 786 (92.4) +PC  ≥55 yrs 2517 (928) –PC  OA:  13365 (96.6%) +PC  2644 (97.5%) –PC  BMI (n, %)  ≤30kg/m2 6225 (45) +PC  ≤30 kg/m2 1238 (45.6) –PC  31 to 35 kg/m2 3582 (25.9) +PC  31 to 35 kg/m2 760 (28) –PC  ≥35 kgn/m2 3539 (25.6) +PC  ≥35 kg/m2 690 (25.4) -PC | Cemented  Pre-coated with PMMA or non-pre-coated  Cruciate-retaining  3135 (22.7%) +PC  626 (23.1%) –PC  Posterior stabilised 10492 (75.8%) +PC  2085 (76.9%) -PC | Tray coating  Diagnosis  Age  Gender  BMI  ASA grade  Femoral coupling design  Surgeon volume  Hospital volume | Mean 2.8 yrs (0 to 7.4) +PC  Mean 1.8 yrs (0 to 7.5) -PC | Revision for any reason other than infection or revision for aseptic loosening of the tibial tray alone | + | Pre-coating HR 2.75, 95% CI 1.34 to 5.65, p=0.006  Posterior stabilisation HR 2.14, 95% CI 1.32 to 3.47, p=0.002  High-flexion implants HR 1.61, 95% CI 1.09 to 2.38, p=0.018  Age >55 yrs HR 0.43, 95% CI 0.27 to 0.67, p<0.001  BMI 30-35 kg (v.<30) HR 1.48, 95% CI 1.00 to 2.19, p=0.050 |
| 6. Blum M et al.  2013  USA^29^ | Prognostic  Level III  Retrospective  Pennsylvania Health Care Cost Containment Council Database  2001 - 2007 | 17385 patients  907 (5.7%) revisions at 5 yrs  6060 (34.9%) male  11325 (65.1%) female  Age:  6107 (35.1%) 18-64 yrs  11278 (64.9%) ≥65 yrs  16436 (94.5%) White  949 (5.5%) African American |  | Race  Age  Sex  Length of hospital stay  Surgical risk of death  Type of health insurance  Hospital surgical volume  Hospital teaching status | 1-5 yrs | All cause | + | 18-64 yrs vs. 65+ yrs HR 2.30, 95% CI 1.96 to 2.69, p<0.0001  Female patients HR 0.81, 95% CI 0.71 to 0.92 , p<0.01  At 5 yrs African American patients HR 1.39, 95% CI 1.08 to 1.80, p=0.01  1 yr mortality 220 (1.3%)  1 yr revision 276 (1.6%); 1.62 95% CI 1.44 to 1.8  5 yr revision 907 (5.7%); 5.71 95% CI 5.37 to 6.07 |
| 7. Bordini B et al.  2009  Italy^32^ | Prognostic  Level III  Retrospective  Register of the Orthopedic Prosthetic Implantology  2000 - 2005 | 9735 TKAs  8892 patients  186 revisions  567 died  2102 (24%) male  6790 (76%) female  Mean age 71.9 yrs (95% CI 71.8 to 72.1)  BMI:  Normal ≤ 25 1840 (18.9%)  Overweight 25-30 4692 (48.2%)  Obese 30-40 3031(31.1%)  Morbidly obese >40 172 (1.8%) | Cemented  Type of insert:  6924 (71.1%) Fixed  2811 (29.9%) Mobile | Age  Gender  Type of insert  BMI or weight | ≥ 18 mos | All cause | + | Mobile insert as compared to fixed insert HR 1.88, 95% CI 1.40 to 2.52, p=0.0001  Age at surgery (for each year) HR 1.05, 95% CI 1.03 to 1.06, p=0.0001  Obesity NS |
| 8. Curtin et al.  2012  USA^55^ | Prognostic  Level III  Retrospective  Medicare 5% Dataset  2001 - 2007 | 61767 TKA  2148 UKA  1112 (1.8%) TKA revision*  34.2% male  65.9% female  Age:  15874 (25.7%) 65 – 69 yrs  18407 (29.8%) 70 – 74 yrs  15998 (25.9%) 75 – 79 yrs  8709 (14.1%) 80 – 85 yrs  2780 (4.5%) 85+ yrs  Race:  56764 (91.9%) Caucasian  3274 (5.3%) African American  1729 (2.8%) Other |  | Age  Gender  Race  Comorbidity  Census region  Year of procedure  Socioeconomic status  Procedure  Surgeon volume | Up to 5 yrs | All cause | + | Older patients (85+) vs. 65-69 yrs 2 yrs HR 0.54, 95% CI 0.37 to 0.79, p=0.002 (TKA and UKA revision)  Older patients (85+) vs. 65-69 yrs 5 yrs HR 0.47, 95% CI 0.33 to 0.67, p< 0.001 (TKA and UKA revision)  Females 2 yrs HR 0.79, p<0.001 (TKA and UKA revision)  Females 5 yrs HR 0.80, p<0.001 (TKA and UKA revision)  Survival rate 2 yrs 98.5%  Survival rate 5 yrs 98.0% |
| 9. Dy et al.  2011  USA^56^ | Prognostic  Level III  Retrospective  Health Benefits Company Database  May 1, 2002 – April 1, 2008 | 10961 TKAs  4056 (37%) male  6905 (63%) female  Mean age 65.2 + 10.7 yrs  Number of preoperative cardiovascular risk factors:  0 – 34.7%  1 – 36.1%  2 – 35.7%  3 – 7.0 %  4 – 0.8% |  | Age  Sex  Type of procedure (THA, TKA, both)  Number of cardiovascular risk factors | Mean 48 mos (24-71 mos)  Note: this follow-up refers to population of THA and TKA | All cause | 0 | No association between cardiovascular risk factors and revision arthroplasty. |
| 10. Fang D et al.  2009  USA^23^ | Prognostic  Level III  Retrospective  1983 - 2006 | 6070 TKAs  3992 patients  51 revisions  1118 (28%) died  1556 (39.0%) male  2436 (61.0%) female  Mean age 70.1 + 8.6 yrs (21 to 93 yrs)  BMI 30.0 kg/m2 (SD 5.5)  Diagnosis:  5803 (95.6%) OA  187 (3.1%) RA  65 (2.1%) osteonecrosis  15 (0.3%) other | Cemented cruciate-retaining, metal-backed nonmodular tibial implant with Anatomically graduated components | Age  Sex  BMI  Overall anatomical alignment  Tibial component position | 6.6 yrs (2 to 22.5 yrs, SD 3.5 yrs) | All cause | 0 | Varus tibial alignment OR 3.0, p=0.04  Overall varus alignment medical collapse OR 6.9, p<0.0001  Mean time to failure 5.5 yrs (SD 3.7) range 6-14 yrs  Survival rate 20 yrs 99%  Older age – no OR reported |
| 11. Fehring T et al.  2004  USA^57^ | Prognostic  Level III  Retrospective  Multi-center  1986 - 1996 | 2091 TKAs  1737 patients  94 revisions  627 (36%) male  1110 (64%) female  Mean age 68 yrs (range 25-96 yrs) | Press-Fit Condylar system  1383 (80%) unilateral  354 (20%) bilateral  1424 (68%) cruciate-retaining  667 (32%) posterior-cruciate-sacrificing | Age  Gender  Size of insert  Style of insert  Thickness of insert  Sheet vendor  Sterilization dose range  Finishing method  Shelf age  Side of surgery  Site of surgery  Posterior cruciate ligament (retained vs. sacrificed) | 5 – 13 yrs; 2023 followed  $\bar{x}$ 5.9 yrs | Wear-related failure | + | Age (years) HR 0.953, 99% CI 0.932 to 0.975, p<0.0001  Males HR 2.771, 99% CI 1.662 to 4.620, p<0.0001  Finishing Method – Wood as compared to polycarbonate-finished HR 17.239, 95% CI 5.957 to 49.890, p<0.0001  Finishing Method – Machined as compared to polycarbonate-finished HR 8.829, 95% CI 3.481 to 22.393, p<0.0001  Sheet Processor B as compared to A HR 7.198, 99% CI 2.017 to 25.686, p<0.0001  Sheet Processor C as compared to A HR 35.859, 99% CI 7.316 to 175.765, p<0.0001  Shelf Age (years) HR 2.867, 99% CI 2.221 to 3.701, p<0.0001  Wear-related failure 108 (5.3%) |
| 12. Furnes O et al.  2002  Norway^58^ | Prognostic  Level III  Retrospective  Norwegian  Arthroplasty Register  1994 - 2000 | 7174 TKAs  213 revisions  1865 (26%) male*  5309 (74%) female*  Mean age 70 yrs (17-93 years)  Diagnosis:  5452 (76%) primary gonarthrosis*  1076 (15%) RA* | 87% cemented  10% hybrid  2% uncemented  65% bicompartmental | Prosthesis brands  Types of fixation  ± patellar resurfacing  Age  Gender  Diagnosis  Use of systemic antibiotic prophylaxis | 0-6.5 yrs | All cause  Revision due to pain  Revision due to infection | + | Bicompartmental (compared to tri-compartmental) HR 0.41, 95% CI 0.18 to 0.93; p=0.03  Survival rate 5 yrs of 6 most commonly used cemented TKAs 95-99% |
| 13. Gioe T et al.  2007  USA^16^ | Prognostic  Level II  Prospective  HealthEast  Joint Registry  Sept. 1991 – Dec. 2005 | 1047 TKAs  Revisions:73  5.6%, 37/653 female  9.2%, 36/394 male  Note: 127 UKA were included in total TKA values with 17 revisions  394 (37.2%) male  653 (62.8%) female  Age 55 and younger – mean 49.8 yrs (28-55 yrs)  977 (93.3%) OA | 5 surgeons >50 surgeries or more  10 surgeons 25-50 surgeries  33 surgeons 25 surgeries or less  738 Cemented  40 Cementless  127 UKA  137 Hybrid | Age  Gender  Pre-operative Diagnosis (OA vs RA vs others)  Cruciate Ligament Status  Component Design  Surgery Year | Mean 55 mos (0-171 mos) | Revision | + | Age, gender, cruciate ligament status and surgical year NS  Cementless vs. cemented HR 2.67, 95% CI 1.27 to 5.63, p=0.01  Hybrid vs. cemented HR 1.83, 95% CI 1.00 to 3.37, p=0.05  Survival rate 14 yrs  - overall 74.5%, 95% CI 67.1 to 81.9  - cemented 84.5%, 95% CI 75.3 to 93.7  - cementless 65.9%, 95% CI 47.9 to 83.9  - <55 yrs 85% |
| 14. Gioe T et al.  2004  USA^59^ | Prognostic  Level III  Prospective  HealthEast  Joint Registry  1991 - 2002 | 5760 TKAs (2.4% bilateral)  168 revisions (including 43 UKA)  2166 (37.6%) male  3594 (62.4%) female  Mean age 68.5 yrs (28 to 95 yrs)  5558 (96.5%) OA*  Note: demographic data contains UKAs | 53 surgeons  Five implant types:  Cemented TKA/all-PE tibia  Cemented TKA/metal-backed tibia  Hybrid TKA  Uncemented TKA  UKA | Age  Gender  Implant type | NR | Revision | + | <70 yrs HR 0.46, 95% CI.0.33 to 0.64, p<0.001  Survival rate 11 yrs overall 93.9%, 95% CI 92.6 to 95.2 |
| 15. Gøthesen Ø et al.  2013  Norway^17^ | Prognostic  Level III  Retrospective  Norwegian Arthroplasty  Register  1994 - 2009 | 17782 TKAs  657 revisions  5612 (31.6%) male  12170 (68.4%) female  Mean age in yrs :  70.0 + 10.0 Profix  70.7 + 9.3 Duracon  69.2 + 10.5 NexGen  69.6 + 9.6 LCS Complete  71.5 +9.0 LCS Classic  71.0 + 9.2 AGC Universal  69.7 + 9.1 AGC Anatomic  Primary OA Diagnosis:  5325 (85.2%) Profix  950 (87.5%) Duracon  674 (86.7%) NexGen  3338 (90.1%) LCS Complete  2268 (87.4%) LCS Classic  1832 (86.9%) AGC Universal  1062 (89.5%) AGC Anatomic | Implants:  Profix  Duracon  NexGen  LCS Complete  LCS Classic  AGC Universal  AGC Anatomic | Implants:  Profix  Duracon  NexGen  LCS Complete  LCS Classic  AGC Universal  AGC Anatomic | 1.8 to 6.9 yrs | Any cause  Aseptic loosening (tibia)  Aseptic loosening (femur)  Dislocation (patella)  Dislocation (other)  Instability  Malalignment  Deep infection  Pain  Polyethylene wear  Stiffness/other | + | Hazard ratio for revision due to all causes compared to Profix:  Duracon HR 2.6, 95% CI 1.9 to 3.4, p<0.001  LCS Classic HR 1.3, 95% CI 1.0 to 1.6, p=0.017  LCS Complete HR 1.5, 95% CI 1.1 to 1.9, p=0.002  AGC Universal HR 1.6, 95% CI 1.3 to 2.0, p<0.001  Hazard ratio for revision due to aseptic tibial loosening compared to Profix:  Mobile-bearing LCS Classic HR 6.8, 95% CI 3.8 to 12.1  LCS Complete HR 7.7, 95% 4.1 to 14.4  Fixed modular-bearing Duracon HR 4.5, 95% CI 1.8 to 11.1  Fixed non-modular bearing AGC universal HR 2.5, 95% CI 1.3 to 5.1  Hazard ratio for revision due to aseptic femoral loosening compared to Profix:  Mobile-bearing LCS Classic HR 2.3, 95% CI 1.1 to 4.8  LCS Complete HR 3.7, 95% 1.6 to 8.9  Fixed modular-bearing Duracon HR 3.4, 95% CI 1.1 to 11.0  Hazard ratio for revision due to malalignment compared to Profix:  Duracon HR 8.7, 95% CI 3.6 to 20.4  Hazard ratio for revision due to deep infection compared to Profix:  Duracon HR 3.7, 95% CI 2.0 to 6.9  LCS Complete HR 2.6, 95% CI 1.6 to 4.3  AGC Universal HR 1.8, 95% CI 1.1 to 3.2  NexGen HR 3.3, 95% CI 1.6 to 6.5  AGC Anatomic HR 2.4, 95% CI 1.2 to 4.7  Hazard ratio for revision due to pain compared to Profix:  Mobile-bearing LCS Classic HR 2.3, 95% CI 1.1 to 4.8  LCS Complete HR 0.4, 95%CI 0.2 to 0.8  AGC Universal HR 2.1, 95% CI 1.5 to 3.0  Hazard ratio for revision due to polyethylene wear compared to Profix:  Duracon HR 16.6, 95% CI 4.9 to 20.4  Survival rate 10 yrs ranged from 89.5% to 95.3% |
| 16. Gøthesen O et al.  Norway  2011^60^ | Prognostic  Level III  Retrospective  Norwegian Arthroplasty Register  2005 - 2008 | 1465 computer-navigated TKAs (CAS)  8214 conventionally operated TKAs (CON)  149 CON revisions  32 CAS revisions  CAS*:  571 (39%) male  894 (61%) female  CON*:  2711 (33%) male  5503 (67%) female    Mean age  CAS 68.8 (95% CI 68.2-69.3) yrs  CON 69.8  (95% CI 69.5-70.0)  Primary gonarthritis  CAS: 1319 (90%)  CON 7310 (89%) | Cemented, uncemented and hybrid TKAs.  Navigation system:  Brainlab  Orthopilot  Stryker  Computer-navigated implants:  AGC: Biomet  Duracon:Stryker  e.motion:Aesculap  LCS Complete:DePuy  Profix:Smith and Nephew | CON vs CAS  Age  Sex  Prosthesis brand  Fixation method  Previous knee surgery  Prepoperative  diagnosis  ASA category | CAS Mean 1.4 yrs  CON Mean 1.8 yrs  Range 1-2 yrs | All Causes | + | CAS RR=1.7, 95% CI 1.1 to 2.5, p=0.02 increased risk of revision than CON  LCS RR=2.1, 95% CI 1.3 to 3.4, p=0.004 increased risk of revision than CON  1 yr survival rate:  CON 98.8% (95% CI 98.6 to 99.0)  CRS 98.5% (95% CI 97.7 to 99.3)  2 yr survival rate:  CON 97.9% (95% CI 97.5 to 98.3)  CAS 96.4% (95% CI 95.0 to 97.8) |
| 17. Harrysson O et al.  2004  USA^44^ | Prognostic  Level III  Retrospective  Swedish Knee Arthroplasty Register  1985 - 1995 | 23195 TKAs  7422 (32%) male*  15773 (68%) female*  33251 >60 yrs  Mean (range) in yrs 55.18 yrs (25.62 to 59.99)  2606 <60 yrs  Mean (range) in yrs 73.21 (60.0 to 95.83)  Diagnosis:  34727 OA  327 osteochondritis  796 posttraumatic arthritis  Note: demographic data contains UKAs | Majority cemented and retained posterior cruciate ligaments | Age  Gender  Year of operation | NR | All cause  Revision attributable to loosening of components  Attributable to infection | 0 | Revision Attributable to Any Reason:  Older patients (≥ 60 yrs) HR 0.49, 95% CI 0.38 to 0.62, p<0.0001  Year of surgery (compared to previous year) HR 0.92, 95% CI 0.89 to 0.96, p<0.0001  Revision Attributable to Loosening of Components:  Older patients (≥ 60 yrs) HR 0.41, 95% CI 0.27 to 0.62, p<0.0001  Year of surgery (compared to previous year) HR 0.87, 95% CI 0.82 to 0.94, p=0.0001  Revision Attributable to Infection:  Year of surgery (compared to previous year) HR 0.91, 95% CI 0.85 to 0.96, p=0.0002  Male HR 1.64, 95%CI 1.23 to 2.18, p=0.0007  Cumulative revision rate 8.5 yrs < 60 yrs 13%  Cumulative revision rate 8.5 yrs ≥ 60 yrs 6% |
| 18. Himanen A et al.  Finland  2007^20^ | Prognostic  Level II  Retrospective  Finnish Arthroplasty  Register  Sept. 1985 – Dec. 1999 | 586 patients with RA  751 TKAs  37 revisions  Moulded:  41 (16%) male  215 (84%) female  Modular:  89 (18%) male  406 (82%) female  Mean age (range) yrs  58 yrs (23 to 80) Moulded  60 yrs (24 to 84) Modular  Mean weight:  66 kg (36 to 101) Moulded  68 kg (37 to 125) Modular | Non-constrained, posterior cruciate-retaining flat on flat components.  Moulded or modular designs  12 surgeons at one institution  Moulded:  142 (55%) cemented  Modular:  318 (64%) cemented | Age  Gender  Weight  Larsen grading  Use of cement  Patella resurfacing  Modular type of tibia component  Tibial slope | Mean 7.9 yrs (0.1 to 14.7) | All cause | + | Age, gender, weight, Larsen grading, use of cement, patella resurfacing, type of tibial component, and tibial slope NS  5 yr cumulative success  - Moulded 96.8%, 95% CI 93.6 to 98.4  - Modular 96.2%, 95% CI 94 to 96.7  10 yr cumulative success  - Moulded 94.4%, 95% CI 90.4 to 96.7  - Modular 93.6%, 95% CI 94 to 97.6 |
| 19. Hooper G et al.  2012  New Zealand^61^ | Prognostic  Level III  Prospective  New Zealand Joint Registry  2005-2008 | 18434 patients  ASA Class:  1 n=2042  2 n= 11669  3 n= 4631  4 n=92  Mean age  ASA class 1: 62.27 yrs  2. 67.82 yrs  3. 70.70 yrs  4. 72.65 yrs |  | ASA Class  Age  Sex | 5 yrs | All cause | + | ASA scores and early revision after TKA NS  Revision rate 1.2 – 1.6% |
| 20. Inacio M et al.  2013  USA^62^ | Prognostic  Level III  Retrospective  Total Joint Replacement Registry  April 2001 –  Dec. 2010 | 62177 TKAs  1362 revisions  2960 (4.8%) died  23349 (37.6 %) male  38828 (62.4%) female  Mean age 68 + 9.3 yrs  BMI (kg/m^2^):  <30 26314 (42.3%)  30-35 18066 (29.1%)  ≥35 16597 (26.7%)  Unknown 1200 (1.9%) | Bearing surfaces:  OZ-CPE  CoCR-HXLPE  CoCr-CPE  HXLPE Inserts:  Durasul  Prolong  Signma | Bearing surfaces  Surgeon  Insert type  Age  Operative type  BMI  Surgeon yearly average volume Hospital yearly average volume  Number of procedures performed by surgeon with specific bearing design | Median 2.8 yrs (IQR 1.2 to 4.9) | All cause  Septic revisions – 1362 (2.2%)  Aseptic revisions – 789 (1.39%) | + | No difference in revision for bearing or inserts  Revision rate 0.689% 95% CI 0.64 to 0.71  Adjusted age, sex, ASA, diabetes, race, BMI, surgical factors |
| 21. Jämsen E et al.  2013  Finland^21^ | Prognostic  Level III  Retrospective  PERFECT database  1998-2008 | 53007 TKAs/pts  1919 revisions  6217 died  15396 (29.0%) male  37611 (71.0%) female  Mean age 70.3 yrs (32-97 yrs)  Comorbidities:  6641 (12.5%) coronary heart disease  3049 (5.8%) atrial fibrillation  2338 (4.4%) heart failure  11025 (20.8%) hypertension  3965 (7.5%) diabetes  2759 (5.2%)  Cancer  4037 (7.6%) pulmonary disease  707 (1.3%) depression  1109 (2.1%) psychotic disorders  934 (1.8%) neurodegenerative disease  25645 (48.4%) any of the above | 50494 (95.3%) Cemented  1543 (2.9%) Hybrid  958 (1.8%)  Cementless  Bilateral 3035 (5.7%) | Age  Sex  Coronary heart disease  Atrial fibrillation  Heart failure  Hypertension  Diabetes  Cancer  Pulmonary disease  Depression  Psychotic disorders  Neurodegenerative disorders | Mean 4.4 yrs (1-4382 days) | All cause | + | ≥1 of diseases analyzed HR 1.23, 95% CI 1.16 to 1.30  Cardiovascular disease HR 1.29, 95% CI 1.14 to 1.45  Psychotic disorders HR 1.41, 95% CI 1.07 to 1.86  Hypertension with early revision (0 – 5 yrs) HR1.14, 95% CI 1.01 to 1.29  Diabetes with early revision (0 – 5 yrs) HR 1.27, 95% CI 1.08 to 1.50  Cancer with late revision (>5 yrs) HR 2.21, 95% CI 1.31 to 3.74  Survival rate 1 yr 98.8%, 95% CI 98.7 to 98.9  Survival rate 3 yr 97.1%, 95% CI 97.0 to 97.2  Survival rate 5 yr 96.3%, 95% CI 96.1 to 96.5  Survival rate 10 yr 94.5%, 95% CI 94.1 to 94.8 |
| 22. Johnson T et al.  2012  USA^63^ | Prognostic  Level III  Retrospective  Health East Joint Registry  Sept. 1991 to Dec. 2009 | 9530 TKAs  627 BKAs  3010 (37%) male  5125 (63%) female  Mean age 67 yrs (range 16-96 yrs) | Cemented | Age  Gender  Length of stay  Year of procedure  Diagnosis  Cruciate status  Patella friendly  Cost of index implant  Follow-up time  PF design | Mean 6.8 yrs (range 0 to 18.8 yrs) | Patella-only revision  Any revision | + | BKAs patella-only revision as compared to TKAs HR 6.9, 95% CI 4.1 to 11.8, p=0.0001  BKAs any revision as compared to TKAs HR 1.67, 95% CI 1.2 to 2.4, p=0.004  Cumulative revision rate for patellar only revisions 4.8% BKA and 0.8% TKA |
| 23. Julin J et al.  2010  Finland^36^ | Prognostic  Level III  Retrospective  Finnish Arthroplasty Register  Jan. 1, 1997 – Dec. 31, 2003 | 32019 TKAs  909 revisions (2.8%)  8986 (28%) male  23051 (72%) female  Age:  ≤55 yrs 1748 (6%)  56-65 yrs 6152 (19%)  >65 yrs 24119 (75%)  Diagnosis:  31042 (97%) primary OA  977 (3%) secondary OA | 9801 (31%) patellar component  29022 (29%) condylar  1799 (9%) constrained condylar  198 hinge designs  1846 (6%) hybrid  817 (3%) cementless  2450 (8%) bilateral | Age  Gender  Reason for primary operation  TKR type  Patellar component  Fixation method | Mean 3.9 yrs (1 to 8 yrs)  Mean time revision 1.9 yrs (1-7.8) | All cause  All cause other than infection (704) | + | For revision for reasons other than infection:  Age ≤ 55 yrs HR 2.9, 95% CI 2.3 to 3.6  Age 56-65 yrs HR 1.7 95% CI 1.4 to 2.0  Patellar component not installed HR 1.4, 95% CI 1.2 to 1.7  Hybrid fixation HR 1.4, 95% CI 1.1 to 1.8  For revision for any reason:  Age ≤ 55 yrs HR 2.4, 95% CI 2.0 to 3.0  Age 56065 yrs HR 1.5, 95% CI 1.3 to 1.7  Secondary OA HR 1.9, 95% CI 1.5 to 2.5  Patellar component not installed HR 1.2, 95% CI 1.1 to 1.4  Hybrid fixation HR 1.4, 95% CI 1.1 to 1.8    5 year survival  - 92% (89.7-93.3)<55 yrs;  - 95% (94.8-96) 56-65 yrs;  - 97% (96.9-97.3) 65+yrs |
| 24. King K et al.  USA  2013^34^ | Prognostic  Level III  Retrospective  Veterans Affairs health Admin  2000 fiscal year | Diabetic cohort:  46-55 yrs 34949 pts  56-65 yrs 70156 pts  66+ yrs 342933 pts  Nondiabetic cohort:  46-55 yrs 800660 pts  56-65 yrs 603918 pts  66+ yrs 1614757 pts |  | Diabetic vs non diabetic cohorts  Age | NR | All Causes | 0 | 46 to 55 yrs diabetic cohort HR 2.9 95% CI 1.5 to 5.8, p=0.004 increased risk of revision as compared to non-diabetic cohort  66+ yrs diabetic cohort HR 1.5 95%, p=0.0037 increased risk of revision as compared to non-diabetic cohort |
| 25. Kreder H et al.  Canada  2003^25^ | Prognostic  Level III  Retrospective  Ontario Health Insurance Plan  April 1992 – March 1996 | 14352 patients  116 revisions after 1 year  282 revisions after 3 years |  | Age  Gender  Comorbidity  Diagnosis  Provider volume  Hospital volume | NR | All cause | + | Low hospital volume (<48) OR 2.23, 95% CI 1.1 to 4.5 at 1 year and OR 1.54, 95% CI 1.0 to 2.4 at 3 years compared to high hospital volume (>113)  Lower patient age per 10 yrs OR 0.77, 95% CI 0.67 to 0.89 at 1 year and OR 0.70, 95% CI 0.66 to 0.81 at 3 years  Surgeon volume, patient comorbidities, diagnosis, gender NS |
| 26. Lygre S et al.  2010  Norway^38^ | Prognostic  Level III  Retrospective  Norwegian Arthroplasty Register  1994 - 2009 | 11887 TKAs  786 revisions  2547 Patella resurfaced  9340 Patella non resurfaced  3328 (28%) male  8559 (72%) female  9628 (81%) OA*  3091 (26%) Previous operation on knee * | Cemented  34% Mobile-bearing prosthesis | Patella replaced (PR)  Non- patella replaced (non-PR)  Age  Sex  Previous operation of the knee  Diagnosis  Prosthesis brand | 9 yrs for patella-resurfaced implants; 7 yrs for implants without patella resurfacing | All cause  Loose femur  Loose tibia  Loose patella  Dislocation, patella  Dislocation, other  Instability  Malalignment  Deep infection  Periprosthetic fracture  Defect polyethylene insert  Pain alone | + | >70 yrs vs. <60 yrs HR 0.4, 95% CI 0.3-0.4, 0<0.001  Previously operated HR 1.3, 95% CI 1.1-1.4, p<0.001  PR compared to non-PR HR 0.84, 95% CI: 0.071-1.0, p=0.052  PR compared to non-PR HR 0.12, 95% CI: 0.06-0.23, p<0.001 for pain alone as reason for revision  PR compared to non-PR HR 1.42 95% CI: 1.03-1.95, p=0.03 for loose tibial components as reason for revision  PR compared to non-PR HR 3.2, 95% CI 1.71-6.11, p<0.001 for defect in polyethylene insert as reason for revision  Compared to reference brand (NR AGC Universal):  NR Tricon RR=1.67, 95% CI 1.24-2.24, p=0.001,  NR Genesis 1 RR=1.43, 95% CI 1.14-1.79, p=0.002,  NR Duracon RR=1.45, 95% CI 1.05-1.99, p=002.  NR Profix RR=0.66, 95% CI 0.52-0.82, p<0.001,  NR e.motion RR=0.09, 95% CI 0.02-0.37, p=0.001,  NR AGC anatomic RR=0.66, 95% CI 0.45-0.99, p=0.04,  PR AGC universal RR=0.48, 95% CI 0.27-0.83, p=0.009,  PR NexGen RR=0.40, 95% CI 0.22-0.74, p=0.004.  Survival rate 15 yrs 92% (91-94) PR TKA  Survival rate 15 yrs 91% (90-92) non-PR TKA |
| 27. Manley M et al.  2009  USA^43^ | Prognostic  Level III  Retrospective  Medicare 5% Sample  Jan. 1, 1997 – Dec. 31, 2004 | 53971 TKAs  1079 (2%) revisions*  All patients ≥ 65 yrs |  | Sex  Age  Race/ethnicity  Arthritis diagnosis  Hospital volume  Surgeon volume  Hospital teaching status  Hospital ownership  Hospital location  Hospital size  Medicare buy-in status | Scheduled at 0.5, 2, 5 and 8 yrs | All cause | + | Lowest volume hospitals (1-25 procedures) at 2 yrs follow-up OR 1.61, 95% CI 0.99 to 2.61, p=0.043  Lowest volume hospitals (1-25 procedures) at 5 yrs  follow-up OR 1.57, 95% CI 1.10 to 2.26, p=0.001  Lowest volume hospitals (1-25 procedures) at 8 yrs follow-up OR 1.52, 95% CI 1.07 to 2.16, p=0.04 |
| 28. McCleery M et al.  2010  United Kingdom^64^ | Prognostic  Level III  Retrospective  Scottish Arthroplasty  Project  April 1985 - April 2008 | 59 288 TKAs  516 early revisions (before 365 days)  2089 late revisions  (after 365 days)  24392 (41%) male  34896 (59%) female  3178 Renal failure  162 Dialysis patients  25 Renal transplant recipients  Renal groups had increased comorbidities (Diabetes mellitus, RA, other inflammatory arthropathy, peripheral vascular disease, steroid) |  | Age  Gender  Comorbidities  Non-renal  Transplant  Dialysis  Renal Failure | < 1 yr | Early revision before 365 days | 0 | Renal dialysis OR 4.175, 95% CI 1.836 to 9.494, p < 0.001  Early revision rate 0.8790 (0.80 to 0.95)  Late revision rate 3.55 (3.38 to 3.6) |
| 29. Namba R et al.  2013  USA^28^ | Prognostic  Level III  Retrospective  Total Joint Replacement Registry  April 2001 -Dec. 2010 | 64017 TKAs  826 revisions (1.3%)  23931 (37.4%) male  40051 (62.6%) female  Mean age 67.3 yrs SD 9.5  5451 (8.5%) bilateral  62194 (97.2%) OA  16836 (26.3%) diabetes  BMI (mean 31.7 kg/m2, SD 6.2)    Race:  3124 (5.2%) Asian  5062 (8.4%) Black  148 (0.2%) Native American  827 (1.4%) Other | 393 surgeons  48 medical centers  Cemented, cementless and hybrid  Bearing surfaces Cobalt Chromium (CoCr) alloy on highly-crosslinked polyethylene, oxinium on conventional polyethylene, CoCr on conventional polyethylene  Fixed cruciate retaining, fixed posterior stabilized, rotating cruciate retaining, rotating posterior stabilized, rotating Low Contact stress | Patient risk factors:  Age  Gender  Race  BMI  ASA score  Type of arthritis  Diabetic status  Bilateral procedures    Implant attributes:  Fixation  Bearing surface  Rotation and stability  Implant flex  Surgeon attributes:  Average yearly volume  Fellowship training    Hospital yearly volume | Mean 2.9 yrs (IQR 1.2 to 4.9 yrs) | Aseptic revision | + | Age (increasing 10 yr increments) HR 0.62, 95% CI 0.57 to 0.67, p<0.001  Race (black vs. white) HR 1.73, 95% CI 1.33 to 2.25, p<0.001  Diabetes HR 1.21, 95% CI 1.04 to 1.41, p=0.014  BMI ≥35 kg/m2 vs. <30 kg/m2 HR 0.78, 95% CI 0.63 to 0.96, p=0.020  Bilateral procedures HR 0.63, 95% CI 0.47 to 0.85, p=0.003  Rotate LCS vs. Fixed PS HR 2.07, 95% CI 1.53 to 2.80, p<0.001  High flexion HR 1.76, 95% CI 1.29 to 2.41, p<0.001  Survival rate 8 yrs 97.6%, 95% CI 97.3 to 97.8 |
| 30. Namba R et al.  2012  USA^27^ | Prognostic  Level III  Retrospective  Community Total Joint Replacement Registry  April 2001 – March 2009 | 47339 TKAs  515 (1.1%) revisions  17705 (37.4%) male*  29634 (62.6%) female*  Mean age 67.5 yrs  OA >95% (45208)  Fixed bearings 41908 (88.5%)  Mobile bearings 4830 (10.2%)  Excluded infected TKAs  Mortality 1858 (3.9%) | Mobile-bearing include Low Contact Stress (LCS), Rotating-Platform Press-Fit Condylar posterior-stabilized (RP PFC PS), and Rotating-Platform Press-Fit Condylar cruciate-retaining (RP-PFC CR)  Fixed bearing include several companies and devices | Bearing type  Age  ASA  BMI  Sex  Race  Diagnosis  Bilateral procedures (4032)  Cruciate-retaining/posterior-stabilized  Surgical approach  Fixation  Patellar resurfacing  Hospital volumes  Surgeon volumes  Fellowship training | NR | Revision other than infection | + | LCS HR 2.01, 95% CI, 1.41 to 2.86, p<0.001  Age (10 yr increments) HR 0.64, 95% CI 0.58 to 0.70, p<0.001  Race: Black vs. white HR 1.82, 95% CI 1.33 to 2.48, p<0.001  Bilateral vs. unilateral HR 0.57, 95% CI 0.39 to 0.83, p=0.003  Unresurfaced patella vs. resurfaced patella HR 2.09, 95% CI 1.07 to 4.06, p=0.03  Survival rate 97.8% (95% CI 97.4 to 98.0) at 6.7 yrs |
| 31. Ong K et al.  2008  USA^45^ | Prognostic  Level II Retrospective  Medicare Claims Data Sample  Jan. 1997 – Dec. 2004 | 65 years and older  N, F/M., Mean age not reported |  | Procedure Duration (median procedure time of 120-150 mins) | 0 to 8 years | Revision | + | TKA primary surgery >240 minutes OR 1.34, 95% CI 1.07 to 1.67, p= 0.012 than <240 minutes  TKA primary surgery between 150 and 180 mins OR 1.31, 95% CI 1.09 to 1.57, p= 0.004 than 120 to 150 mins.  TKA primary surgery <90 minutes OR1.47, 95% CI 1.10-1.95, p=0.008 than 120 to 150 mins |
| 32. Parratte S et al.  2010  USA^65^ | Prognostic  Level III  Retrospective  Mayo Clinic  1985 to 1990 | 398 TKAs with 59 revisions    142 (51%) male  138 (49%) female  Mean age70 ± 8.5 yrs (22 to 89 yrs)  Diagnosis:  337 (85%) OA  30 (8%) RA  31 (8%) Other  Implant Type:  83 (21%) Kinematic Condylar II  114 (29%) Press-Fit Condylar  201 (51%) GENESIS  Unilateral 182 (58%)  Bilateral 118 (42%) | single surgeon  cemented fixation,  all polyethylene patellar component | Mechanical Alignment (mechanically aligned 180 degrees ± 3 degrees versus mechanical axis <177 deg or >183 deg) | 1, 2, 5 years post op and every subsequent 5 years for 15 yrs | Three endpoints:1. Revision for any reason  2. revision because of mechanical failure, aseptic loosening, radiographic wear or patellar complications  3. revision as per 2 with exclusion of patellar complications | 0 | Alignment with revision HR 1.05, p=0.88 NS  Proper mechanical alignment HR1.55, p=0.49 NS  45 (15.4%) revisions in mechanically aligned group  14 (13%) revisions in outlier group |
| 33. Peltola M et al.  2012  Finland^33^ | Prognostic  Level III  Retrospective  Finnish  Arthroplasty  Register  Jan. 1, 1998-  Dec. 31, 2004 | 28 760 TKAs  1000 revisions  7657 (28.2%) male  19448 (71.8%) female  Median age 72 yrs (range 32-97 yrs)  4411 (16.3%) coronary disease  3074 (11.3%) chronic obstructive pulmonary disease and asthma | 34 new femoral and 27 new tibial endoprosthesis models introduced  2283 (8.4%) bilateral  25452 (93.9% cemented  560 (2.1%) cementless  1093 (4.0%) hybrid  8061 (29.7%) patellar resurfacing | Number of operations done with endoprosthesis  Gender  Age  Comorbid diseases | 0 - 5 yrs | All cause | + | First 15 operations with a new endoprosthesis HR 1.48, 95% CI 1.14 to 1.91, p=0.003  Adjusted for age, gender, comorbid disease (COPD and asthma) and surgical characteristics of primary TKA  Coronary disease HR 1.27 95% CI 1.07 to 1.50  COPD and asthma HR 1.27 95% CI 1.06 to 1.51  LOS HR 1.06 95% CI 1.04 to 1.07 |
| 34. Prieto-Alhambra et al.  2011  United Kingdom^42^ | Prognostic  Level III  Retrospective  UK General Practice Research Database  1986 - 2006 | 18726 patients with TKA  215 (1.2%) were revised in non-bisphosphonate users  3 (0.3%) revised in bisphosphonate users  17262 (41.1%) male  24733 (58.9%) female  Mean age 69.98 yrs (SD 9.67)  Mean BMI 28.09 kg/m2 (SD 4.88)  35336 (81.8%) OA of hip or knee  Excluded RA and previous hip fracture  Note: demographics include TKA and THA in study |  | Bisphosphonate use  Age  Sex  BMI  Yr of joint replacement operation  Diagnosis of OA  Previous # before surgery  Use of calcium and vitamin D supplements  Use of hormone replacement therapy or selective oestrogen receptor modulators  Oral glucocorticosteroid treatment  Smoking status and alcohol intake  General practice deprivation score  Location of surgery  Comorbid conditions  Chronic obstructive pulmonary disease  Chronic kidney failure  Neoplasms  Diabetes  Use of drugs that may affect fracture risk | Median 3.5 yrs (IQR 1.6 to 6.3)* | All cause | + | Bisphosphonate use HR 0.40 95% CI 0.15 to 2.07, p=0.068 for implant failure |
| 35. Rand J et al.  2003  USA^19^ | Prognostic  Level II-1  Prospective  Mayo Clinic Total Joint Registry  Jan. 1978 - Dec. 2000 | 8290 participants  11,606 TKAs  3701 (45%) male  4589 (55%) female  Mean age 69 yrs (13 to 102 yrs)  Diagnosis:  10098 (87%) OA  1072 (9%) Inflammatory Arthritis  265 (2%) Post Traumatic Arthritis  125 (1%) Osteonecrosis  46 (<1%) Other | 11166 (96%) Cemented  259 (2%) Uncemented  172 (1%) Hybrid  9 (<1%) Not recorded  4974 (60%) Unilateral  3316 (40%) Bilateral  Design of Patellar Component:  9092 (78%) All-polyethylene  888 (8%) Metal-backed  244 (2%) Not resurfaced  82 (1%) Prior patellectomy  1300 (11%) Not recorded  Design of Tibial Component:  484 (4%) All-polyethylene  8250 (71%) Modular metal-backed  2892 (25%) Nonmodular metal-backed  Implant Type:  8052 (69%) Posterior cruciate-retaining  2994 (26%) Posterior stabilized  120 (1%) Constrained condylar  412 (4%) Posterior cruciate-sacrificing  28 (<1%) Other | Type of implant  Age  Gender  Diagnosis  Type of Fixation  Design of Patellar and Tibial Components | One, two, five years following TKA and every five years subsequently | All Cause | 0 | Implant type  Posterior Stabilized HR 2.6, 95% CI 2.1 to 3.5, p<0.0001 more likely for revision compared to posterior cruciate-retaining  Constrained condylar HR 2.1, 95% CI 0.9 to 4.9, p=0.08 more likely for revision compared to posterior cruciate-retaining  Age  56-70 yrs HR 0.7, 95% CI 0.5 to 0.9, p<0.01ess likely for revision compared to ≤55yrs  >70 yrs HR 0.5, 95% CI 0.3 to 0.6, p<0.0001 less likely for revision compared to ≤55yrs  Gender  Males HR 1.6, 95% CI 1.4 to 2.0, p<0.0001 more likely for revision compared to females  Diagnosis  Inflammatory arthritis HR 0.5, 95% CI 0.3 to 0.7, p<0.001 less likely for revision compared to those with osteoarthritis    Fixation  Uncemented TKAs HR 2.2, 95% CI 1.7 to 3.0, p<0.0001 more likely for revision than cemented  Patella  Metal-backed patellar components HR 2.4, 95% CI 1.9 to 3.1, p<0.0001more likely for revision than all-polyethylene patellar components  Prior patellectomies HR 1.9, 95% CI 1.0 to 3.7, p>0.05 more likely for revision than all polyethylene patellar components  Survival rate 10 yrs 91% (95% CI 90% to 91%)  Survival rate 15 yrs 84% (95% CI 82% to 86%)  Survival rate 20 yrs 78% (95% CI 74% to 81%) |
| 36. Ritter M et al.  USA  2013^41^ | Prognostic  Level III  Retrospective  Single centre  Sept. 1983 – Nov. 2006 | 5342 TKAs  3699 patients  54 revisions  1457 (39.4%) male  2242 (60.6%) female  Mean age 70.2 ± 8.5 yrs (21 to 92 yrs)  Avg BMI prior to surgery 30.0 ±5.5 kg/m2 (range 16.5 to 64.3 kg/m2)  Diagnosis:  5089 (95.3%) OA  178 (3.3%) RA  60 (1.1%) osteonecrosis  15 (0.3%) Other  Avg preoperative alignment 0.1º ± 7.7 º | Cemented, posterior-cruciate retaining  Anatomical Graded Component  Six surgeons  Polyethylene tibial component with cobalt-chromium component – nonmodular block design | BMI  Postoperative tibial alignment  Postoperative femoral alignment  Age | 7.2 ± 3.7 yrs (2 to 22.5 yrs) | Aseptic loosening requiring revision | + | >8º of varus HR 3.4 , p=0.0005 increased risk of revision as compared to 8º to 11º (neutral)  >11º varus HR 2.7, p=0.0082 increased risk of revision as compared to 8 to 11º (neutral) |
| 37. Ritter M et al.  2011  USA^40^ | Prognostic  Level II  Retrospective  Center for Hip and Knee Surgery  Sept. 1983 – Nov. 2006 | 6070 TKAs  3992 patients  54 revisions  1556 (39%) male  2436 (61% ) female  Mean age 70.1± 8.6 yrs (21 to 93 yrs)  Mean Preoperative BMI 30.2 ± 5.6 kg/m2 (16.5 to 64.3 kg/m2)  Diagnosis:  95.6% OA  3.1% RA  1.1% Osteonecrosis  0.2% Other  Mean Preoperative tibiofemoral alignment 0.0º± 7.7º | Six surgeons Anatomically Graduated Components.  Cemented Tibial Component  Posterior-cruciate retaining, metal-backed, nonmodular and composed of cobalt-chromium alloy.  Compression-molded polyethylene liner. | Overall alignment  Component alignments  Preoperative BMI  Age  Sex  Diagnosis  Polyethylene thickness | 7.6 yrs ±3.8 yrs (2 to 22.5 yrs) | Revision for any reason other than infection | 0 | Varus tibial malalignment (<90º) HR 10.6, 95% CI 5.4 to 20.6, p<0.0001 greater risk of failure than those with neutral/valgus alignment    Valgus femoral malalignment (≥8º of valgus) HR 5.1, 95% CI 2.8 to 9.5, p<0.0001 greater risk of failure than those with neutral/varus alignment  Survival rate in well-aligned knees:  BMI 23 to 26 kg/m2 99.3%  BMI ≥41 kg/m2 97.4%  Survival rate in varus knees:  BMI 23 to 26 kg/m2 98.4 %  BMI ≥41 kg/m2 97.1 %  Survival rate in valgus knees:  BMI 23 to 26 kg/m2 99.0%  BMI ≥41 kg/m2 92.9% |
| 38. Robertsson O et al.  1997  Sweden^66^ | Prognostic  Level III  Retrospective  Swedish Knee Arthroplasty  1985 - 1995 | 4381 TKAs  4143 tricompartmental  126 tricompartmental revisions  964 (23%) male  3176 (77%) female  Mean age 66 yrs  All patients with RA | 1925 with patellar components  2214 without patellar components  2781 cemented  273 noncemented | Age  Sex  Index year  Type of Hospital | 10 yrs | All cause | + | 10 yr CRR 5%  Local hospital HR not reported but p<0.001  +/- Patellar component NS |
| 39. Schrama J et al.  2010  Norway^30^ | Prognostic  Level III  Retrospective  Norwegian Arthroplasty Register  Sept. 1989 – June 2008 | 2462 TKAs RA  21832 TKAs OA  176 (0.7%) revisions  6905(29%) male  17389 (71%) female |  | Diagnosis  Age  Sex  Year of Surgery | Not reported | Revision for infection | + | Females HR 0.67, 95% C I 0.47 to 0.88, p=0.006  RA vs. OAH HR 1.6, 95% CI 1.06 to 2.38, p=0.027  RA >5 yrs follow up HR 5.4, 95% CI 1.9 to 16, p 0.002 |
| 40. Stiehl J et al.  2006  USA^24^ | Prognostic  Level III  Retrospective  International Multi-centre  Feb. 23, 1981- Jan. 1, 1997 | 4743 TKAs  259 revisions  1437 (30%) male  3306 (70%) female  Mean age 68 yrs  3666 (77.3%) OA*  901 (19%) RA*  123 (2.6%) post-traumatic | 27 surgeons  10 countries  324 retained both ligaments (RP)  2165 retained posterior cruciate (PCR)  2254 sacrificed both cruciates (BCR)  2838 patella resurfaced  1905 patella unresurfaced  69% cementless | Age  Diagnoses  Gender  Device configuration  Cement status | 5.7 yrs | All cause | + | Females HR 1.513, 95% CI 1.116 to 2..051  Younger patients (for every yr increase) HR 0.979, 95% CI 0.968 to 0.989  OA or Post-traumatic arthritis vs. RAHR 1.839, 95% CI 1.322 to 2.558  PCRs HR 1.552 95% CI 1.157 to 2.081  BCRs HR 2.188, 95% CI 1.454 to 3.294  Patellar resurfacing HR 1.814, 95% CI 1.320 to 2.558  Survival rate 16 yrs 79%, 95% CI 74% to 84 |
| 41. Vessely M et al.  USA  2006^18^ | Prognostic  Level III  Retrospective  Mayo Clinic  Jan. 1987 – Aug. 1989 | 1000 TKAs  745 Patients  45 Revisions (43 pts)  361 (48%) male*  384 (52%) female*  Mean age 70.2 yrs (19-93 yrs)  255 Bilateral  Mean BMI 29.6 kg/m2 (15.2 to 56.4 kg/m2)  Diagnosis:  867 OA  81 inflammatory arthritis (79 RA)  28 failed previous surgery/osteotomy  17 osteonecrosis  7 other | Cemented modular condylar cruciate-retaining TKAs. | Gender  Diagnosis  Age  Tibial insert thickness  BMI  Weight | Mean 15.7 yrs  (14.5 to 17.9 yrs) | All Causes | 0 | Gender, insert thickness, BMI nor patient weight had an association with revision.  5 yr survival rate free of revision for any reason 99.3%, 95% CI 98.7 to 99.8  10 yr survival rate free of revision for any reason 98.6%, 95% CI 97.8 to 99.4  15 yr survival rate free of revision for any reason 95.9, 95% CI 94.3 to 97.5  5 yr survival rate free of revision for mechanical failure 99.5%, 95% CI 99.0 to 99.9  10 yr survival rate free of revision for mechanical failure 99.0%, 95% CI 98.3 to 99.6  15 yr survival rate free of revision for mechanical failure 97.0%, 95% CI 95.6 to 98.4 |

* Numbers calculated from data in article.

Note: If values or variance are not reported, they were not available in the article.

Abbreviations: ASA, American Society of Anesthesiologists; BMI, body mass index; CI, confidence interval; kg, kilograms; HR, hazard ratio; m, metres; mins, minutes; mos, months; NR, not reported; NS, not significant; OA, osteoarthritis; OR, odds ratio; pts, patients; OKS, Oxford Knee Score; RA, rheumatoid arthritis; TKA, total knee arthroplasty; UKA, unilateral knee arthroplasty; yrs, years
